# Prevalence and factors associated with COVID-19 vaccine acceptance in Zambia: a web-based cross-sectional study

**DOI:** 10.11604/pamj.2022.41.112.31219

**Published:** 2022-02-08

**Authors:** Steward Mudenda, Christabel Nang’andu Hikaambo, Victor Daka, Misheck Chileshe, Ruth Lindizyani Mfune, Martin Kampamba, Maisa Kasanga, Margaret Phiri, Webrod Mufwambi, Michelo Banda, Maureen Nkandu Phiri, Moses Mukosha

**Affiliations:** 1 Department of Pharmacy, School of Health Sciences, University of Zambia, P.O. Box 50110, Lusaka, Zambia,; 2 Department of Public Health, Michael Chilufya Sata School of Medicine, Copperbelt University, P.O Box 71191, Ndola, Zambia,; 3 MaryBegg Health Services, 56 Chintu Avenue, Northrise, P.O Box 72221, Ndola, Zambia,; 4 College of Public Health, Zhengzhou University, 100 Kexue Avenue, Zhengzhou, Henan 450001, China,; 5 School of Medicine and Health Sciences, Mulungushi University, P.O Box 80415 Kabwe, Zambia,; 6 HIV and Women´s Health Research Group, University Teaching Hospital, Lusaka, Zambia

**Keywords:** COVID-19 vaccine, vaccination, vaccine acceptability, pandemic

## Abstract

**Introduction:**

vaccinations against COVID-19 have been instituted to contain the pandemic. However, information about the acceptability of COVID-19 vaccines in Zambia is lacking. Therefore, the study assessed the prevalence and factors associated with COVID-19 vaccine acceptance among the general population in Zambia.

**Methods:**

this was an online questionnaire-based cross-sectional study conducted from 13^th^ April to 21^st^ May 2021. We included adult Zambians who had access to Facebook and WhatsApp. A multivariable logistic regression model was fitted to determine factors influencing vaccine acceptability. Data were analysed using Stata version 16.1.

**Results:**

of the 677 participants, only 33.4% (n = 226) would accept the vaccine if made available to them. In multivariable regression analysis, respondents who were older than 41 years compared to the 18 to 23 years age group (aOR: 2.77, 95% CI: 1.03-7.48), those who agreed (aOR; 22.85, 95% CI: 11.49-45.49) or did not know (aOR; 3.73, 95% CI: 2.29-6.07) compared to those who disagreed that the COVID-19 vaccine passed through all the necessary stages to ensure its safety and effectiveness, and those who were aware (aOR; 11.13, 95% CI: 5.31-23.35) compared to those who were not aware that the COVID-19 vaccine reduces virus transmission, were more likely to accept the vaccine. Conversely, entrepreneurs compared to government employees (aOR; 0.24, 95% CI: 0.07-0.79) were less likely to accept vaccination.

**Conclusion:**

awareness of the COVID-19 vaccine was high despite low acceptability levels. These findings are significant as they highlight the need to develop strategies for improving vaccine acceptability in Zambia.

## Introduction

The coronavirus disease 2019 (COVID-19) has remained a public health threat since its first report in December 2019 in Wuhan Province of China [1-4]. It has overburdened healthcare systems globally [3]. Its deleterious effects causing high morbidity and mortality led to the development of vaccines, which are crucial in containing infectious disease outbreaks as they help affected individuals mount early immunological responses and also achieve herd immunity at the population level [5-7].

Vaccines have become integral to limiting the interpersonal, community and global spread of COVID-19 [8, 9]. However, vaccine acceptance and uptake is very crucial in containing pandemics [10]. The refusal of individuals to receive a vaccine made available to them is termed vaccine hesitancy [11-13]. Vaccine hesitancy, specifically against COVID-19, is a global phenomenon impacting many countries, with many myths and beliefs being advanced to support people's hesitancy towards vaccines [14, 15]. This can negate the progress made to ensure an immunised and safe population, especially with an increase in the number of reported variants of severe acute respiratory syndrome coronavirus 2 (SARS-CoV-2) in different countries [16, 17].

The drivers of vaccine acceptance include clear and consistent messages from health authorities, creating an atmosphere of trust, effective vaccine campaigns, confidence in regulatory bodies, and a culturally informed health workforce [10, 18, 19]. However, vaccine availability does not imply uptake due to several factors that may lead to hesitancy such as concerns about side effects and long-term effects on the health of humans [20, 21]. Therefore, disseminating awareness and health information for COVID-19 prevention is vital and could be achieved using various mechanisms, including the media, public talks, and lectures [22]. Some studies have reported positive attitude and access to mass media to be significantly associated with increased awareness of COVID-19 vaccination [23-25].

Despite its immense importance for favourable public health and vaccination program outcomes, vaccine awareness, acceptance and/or hesitancy has not been explored among the general population in Zambia. Therefore, the study assessed the prevalence and factors associated with COVID-19 vaccine acceptability among the general population in Zambia.

## Methods

**Study design, setting and population:** this was an online cross-sectional study done in Zambia's ten provinces (Lusaka, Copperbelt, Central, Western, Southern, Eastern, Northern, North Western, Muchinga and Luapula). Data were collected between 13^th^ April and 21^st^ May 2021. The study was conducted among Zambians who gave informed consent and had access to the online questionnaire. A total of 677 individuals took part in the survey. Due to the ongoing pandemic, the research team identified potential participants through Facebook and WhatsApp.

**Sample size determination:** the sample size was determined using Cochrane´s formula [26]. Acceptability of COVID-19 vaccines of 28% was obtained from a study done in a geographically and socially similar environment, the Democratic Republic of Congo [27]. A 95% confidence level and 5% margin of error were used and resulted in a minimum sample size of 310 participants after accounting for a 10% non-response or incomplete response rate. The final sample size was inflated to account for clustering within the provinces.

**Data collection tool:** the questionnaire was administered to participants electronically via Google forms^®^platform. The data collection tool was adapted from similar African studies and modified to the Zambian setting [27, 28]. Content and face validation of the questionnaire was done by experts from the University of Zambia (UNZA) and Copperbelt University (CBU). The resultant questionnaire was pre-tested on 30 adult Zambians, who were later excluded from the current study. From the initial questionnaire, questions that were based on attitude and perceptions were removed because our study did not focus on these two aspects. The adapted questionnaire was then used to collect data on the participants´ socio-demographic characteristics, awareness, and acceptability of the COVID-19 vaccine. The questionnaire was circulated and reshared every day to maximise responses. In addition, participants were also encouraged to share the questionnaire with their family and friends.

The primary outcome (acceptability) and the secondary outcome (awareness) were assessed on a binary scale (yes/no). These outcomes related to the statements, “If the COVID-19 vaccine was made available, would you accept to be vaccinated?” and “Have you heard of COVID-19 vaccine?”, respectively. The independent variables included age (in years), gender (male/female), marital status (divorced, married, single, widowed), education level (secondary, tertiary), employment (employed, entrepreneur, student, unemployed), source of information about COVID-19 vaccines (family/friends, health workers, internet, radio, television, other), religion (Christianity, Hinduism, Islam, other), areas of residence (rural, urban, rural-urban). In addition, the participants were asked if they knew whether COVID-19 vaccines can reduce SARS-CoV-2 transmission, if the COVID-19 vaccines had passed all the stages of vaccine development to ensure they are safe and effective and if vaccines were only meant for children.

**Statistical analysis:** Stata/IC version 16.1 (Stata Corp., College Station, Texas, USA) was used for our statistical analyses. Categorical variables were presented as frequencies and percentages. The bivariate analysis was performed using the Chi-square test. In cases where the assumption of the Chi-square test was not met (expected cell frequency less than 5), Fisher's exact test was used as appropriate. The primary outcome of the study was the acceptance of COVID-19 vaccines. A binary logistic regression was performed to assess the factors that independently affect COVID-19 vaccine acceptance among the Zambian population. Firstly, potential predictors for acceptance of COVID-19 vaccines were screened using univariable analysis, and variables with p-value < 0.2 were considered in the multivariable binary logistic regression. In the multivariable binary logistic regression model, the participants were dichotomised as would accept vaccines (yes=1, no=0).

The odds ratio (OR) values and 95% confidence intervals (95% CI) were calculated. To account for clustering in the 10 provinces, we calculated the effect measure estimates using robust standard errors. For the final model, interactions between significant predictors were assessed and none reached any statistical significance. Hosmer Lemeshow test was used to assess model fitness. A non-significant Hosmer Lemeshow test indicated a good model fit. Since almost all the participants were aware of COVID-19 vaccines, we could not fit the logistic regression model for this outcome (perfect prediction). A p-value < 0.05 at 95% confidence level was used to indicate statistical significance.

**Ethical approval:** the approval of the study protocol was done by the University of Zambia Health Sciences Research Ethics Committee (UNZAHSREC) under an approval Ref No: 20190217024. The regulatory approval was done by the Zambia National Health Research Authority (NHRA) under an approval Ref No: NHRA00001/12/04/2021. This study is part of an ongoing study on the awareness and acceptance of COVID-19 vaccines among the Zambian adult population. All participants provided consent to take part in the study.

## Results

**Respondents' socio-demographic characteristics:** a total of 677 respondents were included in the analysis. Their socio-demographic characteristics are listed in Table 1. Of the total respondents, 50.7% (n=343) were men and 49.6% (n=336) were married. More than a third of the respondents, 41.2% (n=279) reported television as a source of information. Approximately all respondents, 97.8% (n=662) and 94.9% (n=643) had attained a tertiary level of education and were of Christian faith, respectively.

**Table 1:** characteristics of respondents (N=667)

Characteristics	Frequency	Percentage
**Gender**		
Female	334	49.34
Male	343	50.66
**Age (years)**		
18-23	111	16.40
24-29	187	27.62
30-35	162	23.93
36-41	98	14.48
>41	119	17.58
**Marital status**		
Divorced	10	1.48
Married	336	49.63
Single	319	47.12
Widowed	12	1.77
**Education**		
Secondary	15	2.22
Tertiary	662	97.78
**Employment**		
Government employee	411	60.71
Entrepreneur	40	5.91
Student	161	23.78
Unemployed	65	9.60
**Source of information**		
Family/friends	9	1.33
Health workers	71	10.49
Internet	285	42.10
Radio	21	3.10
Television	279	41.21
Other^a^	12	1.77
**Religion**		
Other^b^	23	3.40
Christianity	643	94.98
Hinduism	2	0.30
Islam	9	1.33
**Areas of residence**		
Rural	68	10.04
Urban	513	75.78
Peri-urban	96	14.18

Key: ^a^None official channels of communication, ^b^Constitutes minor religions in Zambia

**Awareness and acceptance of COVID-19 vaccines:** nearly all the respondents, 99.9% (n=676) were aware of the COVID-19 vaccines. However, less than a third of the respondents, 31.0% (n=210) did not know that the COVID-19 vaccine reduced the transmission of the virus. About 55.8% (n=378) did not think that the COVID-19 vaccine had passed through all stages to ensure its safety. Among the respondents, slightly below one in ten were not aware whether vaccines were only for paediatric use or not. The majority of respondents, 71.8% (324) would refuse vaccination if any COVID-19 vaccine was made available because they were concerned about the safety associated with the vaccine. Approximately 13.3% (38) of respondents were willing to be vaccinated to protect themselves from COVID-19. The summary of the responses to the survey questions on awareness and acceptance of the COVID-19 vaccine are listed in Table 2.

**Table 2:** summary of responses on COVID-19 vaccine questions (N=677)

Question	Frequency	Percentage
**Heard of the COVID-19 vaccine**		
No	1	0.15
Yes	676	99.85
**Would accept COVID-19 vaccine if made available**		
No	451	66.62
Yes	226	33.38
**COVID-19 vaccine reduces virus transmission**		
I don't know	210	31.02
No	153	22.60
Yes	314	46.38
**COVID-19 vaccine has passed through all the necessary stages to ensure its safety**		
I don't know	184	27.18
No	378	55.83
Yes	115	16.99
**Vaccines are only meant for children**		
I don't know	53	7.83
No	613	90.55
Yes	11	1.62
**Reasons for refusal to be vaccinated (n=286)**		
COVID-19 vaccines are not effective	32	7.19
Concerned about the safety of the vaccine	324	71.84
Does not need vaccination	15	3.33
The COVID-19 outbreak in Zambia is not severe	60	13.30
Other reasons	20	4.43
**Reasons for acceptance (n=286)**		
I'm a frontline worker	49	17.13
My employer recommends everyone	12	4.20
To protect myself from COVID-19	38	13.29
Other reasons	187	65.38

**Acceptance of COVID-19 vaccines:** overall, 226 of the 677 respondents would accept to receive the COVID-19 vaccine if made available, giving a prevalence of 33.4% (95% CI: 29.8-37.1). Table 3 shows acceptance levels by respondent characteristics. Of the 226 respondent who would accept vaccination, more than half 57.1% (n=129) were male, 66.4% (n=150) in employment, and 52.7% (n=119) married.

**Table 3:** acceptance of COVID-19 vaccines

Factor	Acceptance		P-value
	No, n (%)	Yes, n (%)	
**Gender**			0.022^a^
Female	237 (52.55)	97 (42.92)	
Male	214 (47.45)	129 (57.08)	
**Age (years)**			0.009^a^
18-23	82 (18.18)	29 (12.83)	
24-29	133 (29.49)	54 (23.89)	
30-35	111 (24.61)	51 (22.57)	
36-41	53 (11.75)	45 (19.91)	
>41	72 (15.96)	47 (20.81)	
**Marital status**			0.161^b^
Divorced	4 (0.89)	6 (2.65)	
Married	217 (48.12)	119 (52.65)	
Single	222 (49.22)	97 (42.92)	
Widowed	8 (1.77)	4 (1.77)	
**Education**			0.997a
Secondary	10 (2.22)	5 (2.21)	
Tertiary	441 (97.76)	221 (97.79)	
**Employment**			0.082^a^
Government employee	261 (57.87)	150 (66.37)	
Entrepreneur	32 (7.10)	8 (3.54)	
Student	115 (25.50)	46 (20.35)	
Unemployed	43 (9.53)	22 (9.73)	
**Source of information**			0.015^a^
Family/friends	6 (1.33)	3 (1.33)	
Health workers	39 (8.65)	32 (14.16)	
Internet	207 (45.90)	78 (34.51)	
Radio	10 (2.22)	5 (2.21)	
Television	173 (38.36)	106 (46.90)	
Other^c^	16 (3.55)	2 (0.88)	
**Religion**			0.038^b^
Other^d^	16 (3.55)	7 (3.10)	
Christianity	432 (95.79)	211 (93.36)	
Hinduism	0	2 (0.88)	
Islam	3 (0.67)	6 (2.65)	
**Areas of residence**			0.857^a^
Rural	47 (10.42)	21 (9.29)	
Urban	339 (75.17)	174 (76.99)	
Peri-urban	65 (14.41)	31 (13.72)	
Overall	N=451 (66.62%)	N=226 (33.38%)	

Key: ^a^Pearson chi-square test; ^b^Fisher's exact test; ^c^None official channels of communication; ^d^Constitutes minor religions in Zambia.

**Factors influencing COVID-19 vaccine acceptance:** the results of univariable and multivariable analysis (binary logistic regression) identified the independent factors that influenced acceptance level, as shown in Table 4. Respondents who were above 41 years old were more likely to accept the COVID-19 vaccine (adjusted odds ratio [aOR]; 2.77, 95% confidence intervals [CI]: 1.03 to 7.48) compared to the 18 to 23 years age group. Those who were entrepreneurs were less likely to accept vaccination (aOR; 0.24, 95% CI: 0.07 to 0.79) than government employees. Respondents who agreed (aOR; 22.85, 95% CI: 11.49-45.49) or did not know (aOR; 3.73, 95% CI: 2.29-6.07) that the COVID-19 vaccine has passed through all the necessary stages to ensure its safety and effectiveness were more likely to accept the vaccine compared to those who disagreed. Similarly, those who were aware that the COVID-19 vaccine reduces virus transmission were more likely to accept vaccination (aOR; 11.13, 95% CI: 5.31-23.35) compared to those who did not.

**Table 4:** univariable and multivariable logistic regression of factors influencing COVID-19 vaccine acceptance

Factor	Crude OR (95% CI)^b^	P-value	Adjusted OR (95% CI)^c^	P-value
**Age (years)**				
18-23	Ref		Ref	
24-29	1.15(0.68-1.95)	0.609	0.79(0.37-1.69)	0.544
30-35	1.30(0.76-2.22)	0.340	1.00(0.39-2.58)	0.999
36-41	2.40(1.34-4.29)	0.003	2.56(0.93-7.09)	0.070
>41	1.85(1.05-3.23)	0.032	2.77(1.03-7.48)	**0.045**
**Employment**				
Government employee	Ref		Ref	
Entrepreneur	0.44(0.20-0.97)	0.042	0.24(0.07-0.79)	**0.019**
Student	0.70(0.47-1.03)	0.073	1.30(0.61-2.78)	0.491
Unemployed	0.89(0.51-1.55)	0.680	0.83(0.35-1.96)	0.681
**Religion**				
Othera	Ref		Ref	
Christianity	1.11(0.45-2.76)	0.811	0.80(0.22-2.97)	0.745
Islam	4.57(0.88-23.71)	0.070	8.13(0.84-78.80)	0.071
**COVID-19 vaccine reduces virus transmission**				
No	Ref		Ref	
Yes	20.51(10.40-40.45)	<0.001	11.13(5.31-23.35)	**<0.001**
I don't know	2.48(1.17-5.22)	0.017	1.81(0.81-4.80)	0.151
**COVID-19 vaccine has passed through all the necessary stages to ensure its safety and effectiveness**				
No	Ref		Ref	
Yes	40.88(22.09-75.65)	<0.001	22.85(11.49-45.49)	**<0.001**
I don't know	4.03(2.67-6.10)	<0.001	3.73(2.29-6.07	**<0.001**
**Vaccines are only meant for children**				
No	Ref		Ref	
Yes	5.08(1.33-19.35)	0.003	2.56(0.41-15.95)	0.314
I don't know	0.29(0.13-0.65)	<0.001	0.38(0.15-1.01)	0.051

Key: OR-odds ratio, 95% CI- 95% confidence intervals, ^a^Constitutes minor religions in Zambia ^b^Calculated from univariable logistic regression of the effect of each factor on odds of vaccine acceptance. ^c^Calculated from multivariable logistic regression of the combined effect of all factors (that had p<0.2 at bivariate analysis) on vaccine acceptance.

## Discussion

The study was conducted to assess the prevalence and factors associated with COVID-19 vaccine acceptability among the general population in Zambia. Although 99.9% of the participants were aware of the COVID-19 vaccine, only about 33.4% were willing to accept the vaccine if made available to them. Factors influencing vaccine acceptability included advanced age, being an entrepreneur than a government employee, knowing that the COVID-19 vaccine has passed through all the necessary stages and is safe, effective and reduces virus transmission.

The acceptance of the COVID-19 vaccine among the adult Zambian population was lower than that reported in Ethiopia [29], Jordan [30], the Middle East population [31] and Australia [32]. The low acceptance level found in our study can be attributed to the fact that the participants were sceptical about the COVID-19 vaccine's potential adverse effects and effectiveness. Besides, the lower number of confirmed COVID-19 infections in Zambia during the study period could have also contributed to vaccine hesitancy. Higher rates of acceptance of COVID-19 vaccines were reported in other studies due to the high severity of the pandemic in those countries and the fear of contracting the virus [33]. In areas of high COVID-19 morbidity and mortality rates, individuals are likely to receive the COVID-19 vaccine so that they do not contract the disease [33, 34]. Slightly similar acceptability levels of COVID-19 vaccines have been reported in other countries. For example, an acceptability level of 28.8% was reported in France [35], 28% in the Democratic Republic of Congo [27]. The reasons for the lower acceptability level in these studies were concerns about the safety and effectiveness of COVID-19 vaccines. The lower acceptance of COVID-19 vaccines reported in our study and similar studies may affect the purpose of COVID-19 vaccination programs. Having good immunity against a particular infectious agent within a population is necessary to limit the spread of the pathogen causing the infection [14, 36, 37]. This immunity would be achieved through vaccination. However, with the vaccine acceptance level observed in our study and other studies, it would be difficult to stop the transmission of the virus within the community.

Regarding age, the majority of respondents in the current study above the age of 41 years were willing to be vaccinated so that they are protected from COVID-19. A similar study in Libya reported that individuals aged 41-50 years were willing to receive the vaccine [38]. However, a similar study in Malaysia reported that people older than 60 years were likely to refuse vaccination against COVID-19 [39]. Individuals that receive the correct or adequate information about the benefits of vaccines are likely to receive the COVID-19 vaccine, compared to those who receive misinformation or insufficient information. Further, our findings indicated that entrepreneurs were more likely to accept the COVID-19 vaccine than those government employees. Evidence has shown that employees are likely to receive a COVID-19 vaccine if their employer recommended it [33]. Our current study findings indicated that many individuals who knew that COVID-19 vaccines reduce the transmission of the virus were more likely to accept the COVID-19 vaccine. These findings corroborate the findings from a similar study on COVID-19 acceptance [39]. Therefore, individuals with a lack of information on the benefits of vaccination against COVID-19 are less likely to accept the vaccine.

Consistent with other published studies [27, 28, 39, 40], we found that respondents concerned about vaccines' potential adverse effects and effectiveness are less likely to accept vaccination. In the current study, despite the availability of COVID-19 vaccines, many participants (66.6%) were unwilling to be vaccinated. Many participants felt that the COVID-19 vaccines had potential adverse effects. The fear of potential adverse effects has been reported to be one of the major reasons leading to vaccine hesitancy [32, 33, 41]. For this reason, there is a need for health authorities to provide adequate information to the general population on the adverse effects of vaccines before they are rolled out. Many individuals were hesitant to be vaccinated because the COVID-19 epidemic has been less severe in Zambia and many African countries [42-44]. Similarly, a study done in Taiwan, where COVID-19 was less severe, also reported low vaccine acceptance [45]. The participants in our study were unwilling to be vaccinated because of concerns about the effectiveness of vaccines. Earlier studies on vaccines have shown that individuals feel that vaccines are not effective [46, 47]. This lack of trust in vaccines has led to an increased unwillingness to receive the COVID-19 vaccines [41, 48, 49]. Distrust in vaccines´ effectiveness could be a result of infodemics, misconceptions, and rumours of immunised people later contracting the disease [50]. Therefore, there is a need to educate the general population about vaccines and their development stages before being administered to humans. The government and health authorities need to educate the public on the benefits of vaccines and the need for vaccinations.

**Strengths and weaknesses of the study:** to the best of our knowledge, this is the first study on COVID-19 vaccine awareness and acceptability among the general population in Zambia. Our findings could help inform the development of strategies to address vaccine hesitancy and increase the uptake of vaccinations among the Zambian population and other sub-Saharan African settings. Moreover, this study highlights the factors that need to be addressed if COVID-19 vaccination programs are to be successful. However, the study had few limitations. Many Zambians who have no access to the social media platforms that were used to collect data did not participate in the study; hence, the generalisation of our findings may not apply to the entire Zambian population, possibly a risk factor resulting in non-response bias. In the analysis, potential confounders were adjusted using a multivariate logistic regression model.

**Unanswered questions and future research:** drivers of vaccine hesitancy and/or acceptance in technologically disadvantaged and rural poor populations in Zambia. The incidence of adverse events among vaccinated individuals and implications for future participation in vaccination programs.

## Conclusion

The COVID-19 vaccine acceptance was low among the adult Zambian population, while awareness was high. This indicates the unwillingness of the majority of Zambians to be vaccinated against COVID-19. The findings of this study can be used to formulate and implement strategies that can help improve the willingness of individuals to be vaccinated. Also, despite being an online survey, the findings of this study can be used to prepare individuals for vaccinations in future epidemics.

### What is known about this topic

- COVID-19 is a public health problem that has negatively affected the globe;
- COVID-19 vaccines have been developed and are being administered to contain the pandemic;
- Potential adverse effects and concerns about the effectiveness of COVID-19 vaccines are among the factors leading to vaccine hesitancy.

### What this study adds

- This study adds information that can be used to address vaccine hesitancy during pandemics;
- Increasing COVID-19 vaccine acceptance will lead to increased uptake and achievement of herd immunity;
- Dissemination of information about the COVID-19 vaccines is more trusted by individuals when it comes from healthcare workers.
